# Near-Infrared Fluorescence for Precision Breast Surgery in Nonpalpable Lesions: A Case Report

**DOI:** 10.7759/cureus.94937

**Published:** 2025-10-19

**Authors:** Sebastian Alba Posse, Fernando Dip, Rene Aleman, Alberto Rancati, Diego Sinagra

**Affiliations:** 1 Department of General Surgery, University of Buenos Aires, Buenos Aires, ARG; 2 Heart, Vascular, and Thoracic Institute, Cleveland Clinic Florida, Weston, USA

**Keywords:** breast conserving surgery, breast neoplasms, fluorescence imaging, indocyanine green, nonpalpable breast lesions

## Abstract

Accurate localization of non-palpable breast lesions remains challenging in clinical practice, and conventional methods carry procedural and patient-comfort limitations. Near-infrared (NIR) fluorescence imaging via indocyanine green (ICG) provides an innovative approach as a surgical adjunct for real-time, intraoperative visualization of target anatomy. In the context of performing safe surgery, fluorescence-guided surgery (FGS) effectively delivers a safe, feasible, and reproducible technique to localize non-palpable breast nodules, sparing the nuisances of suboptimal contemporary alternatives.

This is the case of a 47-year-old woman with a BI-RADS 4, non-palpable left breast lesion, who underwent ultrasound-guided injection of a modified ICG solution immediately before surgery. Real-time fluorescence provided delineation of the lesion, and guided skin incision, dissection trajectory, and depth control. This approach underscores the technical feasibility, workflow integration, and intraoperative decision support conveyed by FGS in non-palpable breast lesions. The favorable outcomes of the present case report align with superior outcomes reported in the literature. Nonetheless, a larger, systematic comparison with established localization techniques is warranted to define indications, accuracy, and resource implications for broader surgical adoption.

## Introduction

Advances in diagnostic strategies have improved the detection of nonpalpable breast lesions, with the combined use of mammography and ultrasound reaching a sensitivity of 97.3% [1]. Approximately 3% of women in primary care present with a breast complaint, and fewer than 10% of these result in malignancy [2]. Breast cancer remains the leading cause of cancer in women worldwide, and prognosis is critically dependent on the stage at which the disease is diagnosed, underscoring the importance of early detection and timely intervention [3]. Given the clinical profile of nonpalpable breast tumors, breast-conserving surgery (BCS) is the standard of care for patients with localized, early-stage breast cancer, offering superior survival outcomes to mastectomy while preserving cosmesis and reducing postoperative psychological burden [4]. The cornerstone of BCS is the safe and accurate removal of tumors while preserving breast cosmesis and minimizing postoperative complications [5]. Current localization techniques for nonpalpable breast tumors include radioactive occult lesion localization (ROLL), radioactive seed localization (RSL), intraoperative ultrasound-guided localization (USGL), intraoperative supine magnetic resonance imaging (SMRI), anchor-guided localization (AGL), and cryo-assisted localization (CAL). Despite the multiple alternatives, recent literature shows heterogeneous outcomes regarding margin positivity, reoperation rates, and complication rates, with results akin to those of wire-guided localization (WGL) [6]. 

Accurate intraoperative localization is essential to the success of BCS, ensuring safe and complete surgical excision. Fluorescence-guided surgery (FGS) with indocyanine green (ICG) has emerged as a promising adjunct, offering accuracy, feasibility, and safety for intraoperative localization of nonpalpable breast tumors. Its efficacy appears equivalent to other localization methods and may reduce rates of tumor-positive margins [7]. Although current literature remains limited, FGS has demonstrated encouraging results. In a multicenter, randomized, open-label, parallel phase 3 clinical trial across four centers, FGS improved histopathological accuracy and reduced skin pigmentation rates [8]. Similarly, a single-center randomized controlled trial reported significantly higher rates of clear margins in nonpalpable breast cancer excision with ICG, versus the conventional WGL technique [9]. The present case report highlights the integration of FGS in the guided, accurate excision of a nonpalpable breast tumor.

## Case presentation

This is the case of a 47-year-old woman with no significant past medical history, referred to a specialized breast clinic after an abnormal finding was noted on screening imaging. Preoperative diagnostic mammography and ultrasound identified a 9 mm hypoechoic nodule in the upper outer quadrant of the left breast, classified as BI-RADS 4. Clinical examination confirmed the lesion was non-palpable. A core needle biopsy established a diagnosis of ductal carcinoma in situ (DCIS), and the patient was scheduled for surgical excision.

To enhance intraoperative localization, a fluorescence-guided approach utilizing ICG in combination with macromolecular carriers was performed. A modified reconstitution of ICG with 5 mL of sterile water was prepared, and 1 mL of this solution was combined with a 1 mL vial of lyophilized macroaggregated albumin. The preparation was handled with care to preserve protein macromolecular integrity, thereby improving retention within the breast tissue [8].

**Figure 1:**
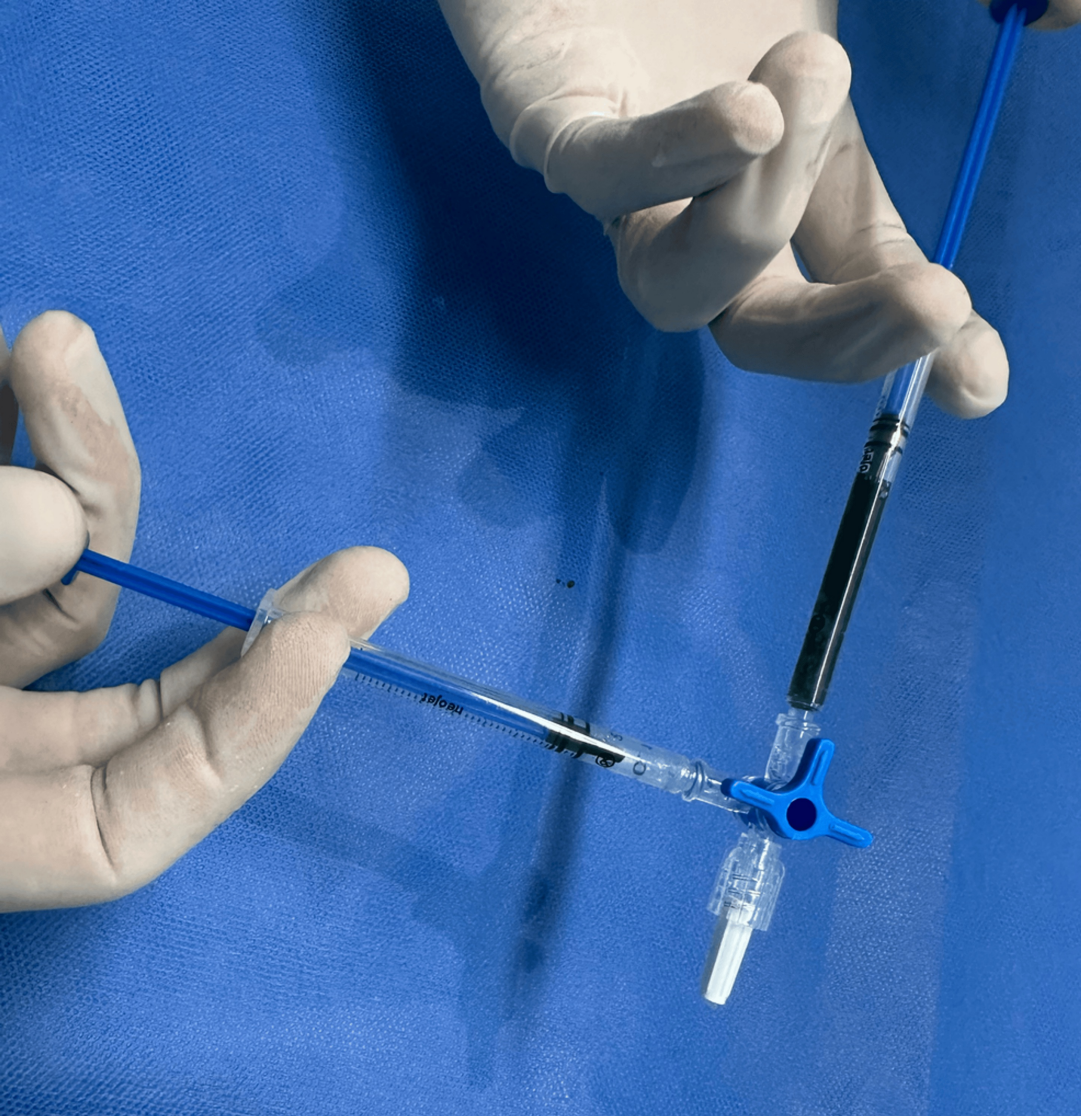
Modified indocyanine green solution with macromolecular carriers The modified solution was prepared immediately before low-dose, ultrasound-guided administration.

From this mixture, 0.6 mL was further diluted with hyaluronic acid to increase viscosity and prolong tissue anchorage. A total of 1 mL of the final solution was injected directly into the lesion under real-time ultrasound guidance, and a guided surgical excision was performed (Figure 1). The patient was provided with verbal and written consent prior to the intervention.

Surgical technique

**Figure 2:**
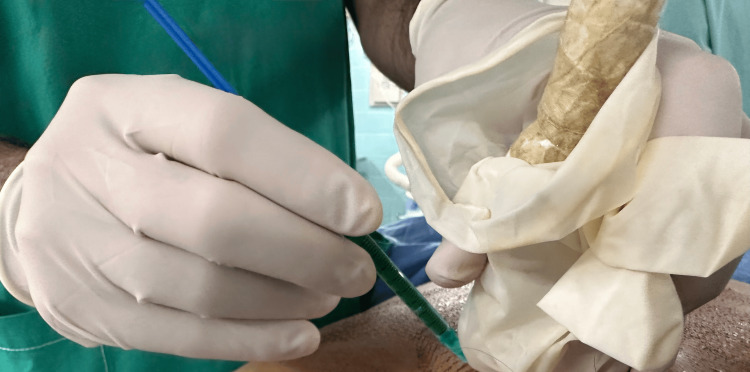
Ultrasound-guided modified dye injection 1 mL of the prepared solution was injected at the level of the target lesion.

The procedure was carefully planned to ensure clear oncologic margins, maximize histopathological accuracy, and reduce the risk of disease progression or reintervention. Following induction of general anesthesia, ultrasound-guided administration of the modified ICG solution was performed, with injection at the level of the target lesion. A gentle, clockwise massage was applied to facilitate homogeneous distribution of the dye within the surrounding breast tissue (Figure 2).

**Figure 3:**
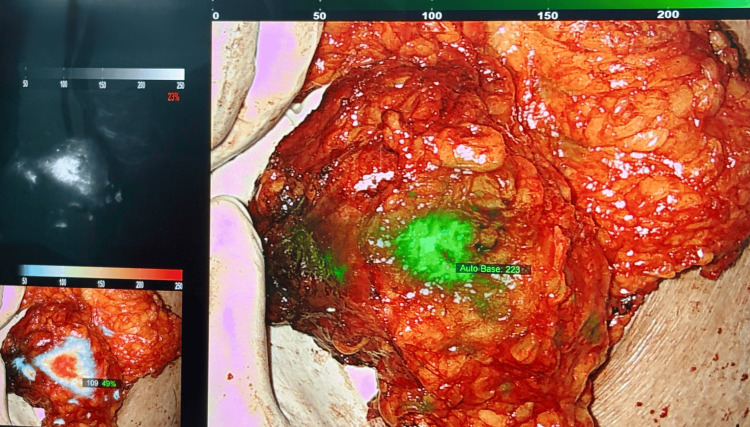
Fluorescence-guided imaging assessment of the excised breast lesion Intraoperative fluorescence-guided imaging allowed for guided dissection in multiple visualization modes to comprehensively excise all target tissue.

A handheld fluorescence imaging device (EleVision™ IR; Medtronic, Fridley, MN, USA) was prepared in sterile fashion on the back table at the time of dye injection. The patient was positioned supine, with the ipsilateral arm abducted. A strategically placed 4 cm arciform axillary incision was selected to optimize both oncologic adequacy and cosmetic outcomes. Skin flaps were then raised to expose the lesion. Fluorescence guidance was introduced intraoperatively to assist in dissection. The imaging system was utilized in multiple visualization modes, including overlay, contrast, and color segmentation, to evaluate the intensity and distribution of the fluorescent signal. This enabled precise delineation of the lesion and surrounding margins (Figure 3).

A lumpectomy was subsequently performed, and the excised specimen, measuring 9 × 5 × 5 mm, was oriented and inked for pathological assessment. To restore breast contour, a dermoglandular plane was carried out using both superficial and deep dissection, addressing the tissue void created by excision. Titanium clips were placed within the lumpectomy cavity to facilitate accurate targeting for adjuvant radiation therapy, if deemed necessary. The skin was closed with interrupted 3-0 Prolene sutures, ensuring precise edge eversion and tension-free approximation. The procedure was completed without complications. The patient tolerated surgery well and was transferred to the post-anesthesia care unit in stable condition to continue the expected postoperative recovery.

Postoperative care

**Figure 4:**
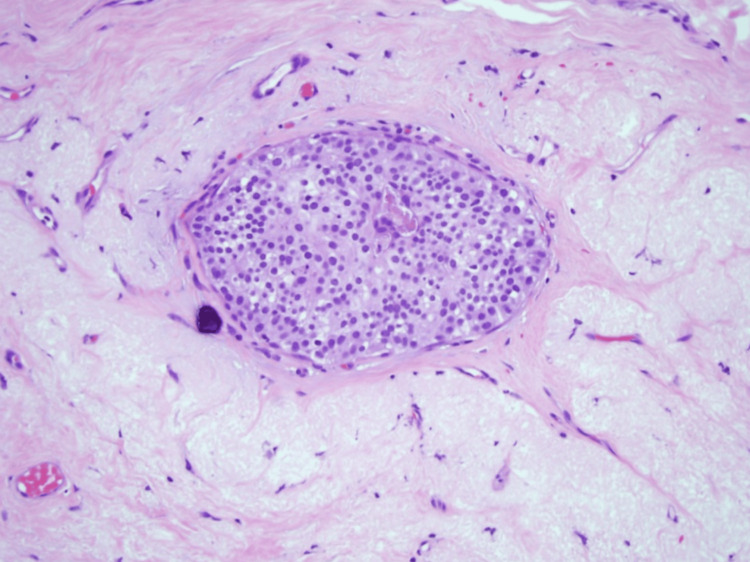
Postoperative histopathological assessment Histopathology evaluation confirmed the presence of low-grade ductal carcinoma in situ (DCIS) without evidence of invasion (Tis (DCIS), N0, M0).

Postoperative pathological evaluation confirmed the presence of low-grade DCIS without evidence of invasion (Tis (DCIS), N0, M0) (Figure 4). Fluorescence-guided imaging facilitated the precise localization of the lesion, enabling direct segmental excision. Despite the absence of quantitative metrics linking fluorescence intensity to target tissue, the use of advanced fluorescence visualization modes provided highly reliable, real-time guidance for precise resection - a result further validated by postoperative histopathology confirmation. No diffusion of dye was observed in the adjacent breast parenchyma or sentinel lymph nodes, supporting the accuracy of intraoperative visualization achieved with the modified ICG solution. The patient was discharged in stable condition 24 hours after surgery and proceeded with an uneventful recovery under routine clinical follow-up.

## Discussion

Accurate tumor localization is essential for achieving oncologic safety while preserving cosmesis in BCS. WGL has long been the standard technique, yet it is associated with several disadvantages, including the need for same-day performance due to risks of wire migration or withdrawal, patient discomfort, and interference with surgical excision technique [10-12]. These limitations have prompted the development of multiple alternatives, including ROLL, RSL, USGL, and magnetic- or carbon-based methods. Among these, USGL and AGL have demonstrated non-inferiority to WGL in the localization of nonpalpable breast cancers, with evidence suggesting lower positive margin and reoperation rates [6]. FGS with ICG continues to trail as a promising adjunct for intraoperative visualization of nonpalpable breast lesions. Recent studies evaluating modified ICG formulations and ICG-based composites suggest improved intraoperative visualization and resection accuracy. While feasibility and safety are well established, claims of superiority should be interpreted cautiously until validated through standardized comparative trials [7-9,13,14]. 

The use of macromolecular carriers, such as macroaggregated albumin, enhances the retention of ICG within breast tissue, while the addition of hyaluronic acid increases viscosity, prolonging localization stability. These mechanistic modifications theoretically reduce dye diffusion and improve intraoperative accuracy, although direct quantitative validation remains limited across all surgical specialties integrating FGS. A phase-2 clinical trial reported that ICG-hyaluronic acid can be used for accurate preoperative localization without skin pigmentation in benign breast disease [15]. Furthermore, in a multicenter, randomized, open-label phase 3 trial, the same formulation demonstrated statistically significant improvements in histopathological accuracy (0.26 ± 0.13 vs. 0.33 ± 0.17, p = 0.01) and a markedly lower rate of skin pigmentation (0.00% vs. 30.77%, p < 0.01) compared with activated charcoal, with no dye-related adverse events [8]. In the present case, FGS facilitated precise lesion identification and complete excision without the drawbacks associated with WGL. This approach enhances intraoperative optics for small-caliber lesions that are otherwise challenging to delineate under conventional white light. Given the broad clinical applications of ICG - including angiography, tumor mapping, and sentinel lymph node identification - its integration into BCS may streamline multiple imaging needs within a single surgical workflow [16-19]. 

Emerging clinical data increasingly support the utility of FGS in breast surgery, with studies reporting margin clearance rates that are comparable to or superior to conventional methods. However, variations in study design, sample size, and fluorescence signal quantification methods warrant careful interpretation of pooled outcomes. Reports have also associated ICG localization with improved lesion centralization and reduced resection volumes - factors that are critical for preserving cosmetic outcomes, minimizing postoperative pain, and reducing psychological burden after lumpectomy [7,13,14]. A systematic review, including 11 studies and 366 patients, confirmed that ICG is accurate, safe, and effective for localizing nonpalpable breast tumors. Importantly, comparisons with WGL and RSL showed equivalent localization accuracy while avoiding patient discomfort, logistical challenges, and exposure to radioactive agents. Furthermore, ICG localization achieved higher rates of negative margins (up to 90.5% vs. 83.3% with ultrasound guidance) and reduced specimen volumes [7]. 

Altogether, this report highlights the growing clinical relevance of combining ultrasound imaging with fluorescence-guided dissection and lymphography to provide a versatile, minimally invasive alternative to conventional localization strategies. Nevertheless, the interpretation of fluorescence intensity remains subjective and lacks standardized thresholds, limiting reproducibility across settings. The authors acknowledge that the level of evidence inherent to a case report restricts the generalizability of conclusions. Future research should prioritize multicenter, randomized trials with consistent methodology, standardized fluorescence quantification, and long-term oncologic and cosmetic follow-up. These studies will be pivotal in determining whether ICG-based FGS can evolve from an experimental adjunct to an evidence-based standard for nonpalpable breast lesion localization. 

## Conclusions

This case highlights the feasibility of fluorescence-guided localization using ICG in combination with macromolecular carriers as a reliable alternative for the excision of nonpalpable breast lesions. The technique enabled precise lesion targeting, demonstrated seamless integration into the operative workflow, and excellent patient tolerance. While these findings underscore the potential of FGS to enhance accuracy in BCS, larger prospective studies are needed to validate its clinical utility and to define its role within standard surgical practice.
